# Ligand-based virtual-screening identified a novel CFTR ligand which improves the defective cell surface expression of misfolded ABC transporters

**DOI:** 10.3389/fphar.2024.1370676

**Published:** 2024-04-11

**Authors:** Shogo Taniguchi, Francois Berenger, Yukako Doi, Ayana Mimura, Yoshihiro Yamanishi, Tsukasa Okiyoneda

**Affiliations:** ^1^ Department of Biomedical Sciences, School of Biological and Environmental Sciences, Kwansei Gakuin University, Nishinomiya, Japan; ^2^ Graduate School of Frontier Sciences, The University of Tokyo, Kashiwa, Japan; ^3^ Department of Complex Systems Science, Graduate School of Informatics, Nagoya University, Graduate School of Informatics, Nagoya University, Nagoya, Japan

**Keywords:** CFTR, ligand, ABCB1, in silico screening, cystic fibrosis

## Abstract

Cystic fibrosis (CF) is a monogenetic disease caused by the mutation of CFTR, a cAMP-regulated Cl^−^ channel expressing at the apical plasma membrane (PM) of epithelia. ∆F508-CFTR, the most common mutant in CF, fails to reach the PM due to its misfolding and premature degradation at the endoplasmic reticulum (ER). Recently, CFTR modulators have been developed to correct CFTR abnormalities, with some being used as therapeutic agents for CF treatment. One notable example is Trikafta, a triple combination of CFTR modulators (TEZ/ELX/IVA), which significantly enhances the functionality of ΔF508-CFTR on the PM. However, there’s room for improvement in its therapeutic effectiveness since TEZ/ELX/IVA doesn't fully stabilize ΔF508-CFTR on the PM. To discover new CFTR modulators, we conducted a virtual screening of approximately 4.3 million compounds based on the chemical structures of existing CFTR modulators. This effort led us to identify a novel CFTR ligand named FR3. Unlike clinically available CFTR modulators, FR3 appears to operate through a distinct mechanism of action. FR3 enhances the functional expression of ΔF508-CFTR on the apical PM in airway epithelial cell lines by stabilizing NBD1. Notably, FR3 counteracted the degradation of mature ΔF508-CFTR, which still occurs despite the presence of TEZ/ELX/IVA. Furthermore, FR3 corrected the defective PM expression of a misfolded ABCB1 mutant. Therefore, FR3 may be a potential lead compound for addressing diseases resulting from the misfolding of ABC transporters.

## 1 Introduction

Cystic fibrosis transmembrane conductance regulator (CFTR) is a membrane protein located at the apical plasma membrane (PM) of epithelial cells in the respiratory and gastrointestinal tracts. Belonging to the ATP-binding cassette (ABC) transporter family, it functions as a cAMP-regulated anion channel, facilitating the transport of ions, including chloride (Cl^−^) and bicarbonate ions (HCO3^-^) (Riordan et al., 1989). CFTR comprises two membrane-spanning domains (MSD1, 2), two cytosolic nucleotide-binding domains (NBD1, 2), and a regulatory (R) domain (Riordan, 2008). Mutations in the CFTR gene cause cystic fibrosis (CF), a recessive genetic disorder (Riordan, 2008). Among these mutations, the ΔF508 mutation occurring in the NBD1 domain is the most prevalent, accounting for approximately 90% of CF cases. The ΔF508 mutation results in folding deficiencies in CFTR within the endoplasmic reticulum (ER). Consequently, the misfolded ∆F508-CFTR undergoes ubiquitination and premature degradation through ER-associated degradation (ERAD), leading to compromised maturation and reduced expression at the PM (Jensen et al., 1995; Ward et al., 1995; Okiyoneda and Lukacs, 2012). The ΔF508 mutation causes at least two structural abnormalities in CFTR. Firstly, it destabilizes NBD1 due to the deletion of phenylalanine at position 508 (Rabeh et al., 2012). Secondly, it disrupts the normal interdomain interaction between NBD1 and MSD (Mendoza et al., 2012; Rabeh et al., 2012). Stabilizing ΔF508-NBD1 with a suppressor mutation and correcting the abnormal interdomain interaction between NBD1 and MSD have shown promising results in restoring ΔF508-CFTR PM expression levels, approaching those of the wild-type CFTR, enhancing functionality by up to 65%–80% (Rabeh et al., 2012).

The folding defects observed in ∆F508-CFTR can be partially rectified through different approaches. Low-temperature incubation (26°C–30°C) has been shown to mitigate the folding issues associated with CFTR mutants like ∆F508-CFTR (Denning et al., 1992). Additionally, small molecule compounds known as CFTR correctors play a pivotal role in rescuing the folding, processing, and trafficking of CFTR mutants that are retained within the ER due to misfolding (Pedemonte et al., 2005; Van Goor et al., 2011). These correctors, acting as pharmacological chaperones, have been identified through cell-based high-throughput screenings and *in silico* (virtual) screenings based on the CFTR structure (Pedemonte et al., 2005; Van Goor et al., 2011; Odolczyk et al., 2013; Phuan et al., 2014; Veit et al., 2018; Orro et al., 2021; Fossa et al., 2022). Pharmacological chaperones, including ligands and substrates, directly engage with their target proteins, often leading to an increase in thermodynamic stability (Bernier et al., 2004; Arakawa et al., 2006; Loo and Clarke, 2007). Structural studies have confirmed the binding sites of certain corrector compounds (Fiedorczuk and Chen, 2022).

The rational use of corrector combination therapy has been proposed based on their mode of action (Okiyoneda et al., 2013; Veit et al., 2018). Trikafta, a triple combination CF drug containing two CFTR correctors, Tezacaftor (TEZ, VX-661) and Elexacaftor (ELX, VX-445), along with the CFTR potentiator Ivacaftor (IVA, VX-770) facilitating channel opening (Van Goor et al., 2009), received approval in 2019 (Keating et al., 2018). Trikafta has shown significant effectiveness in correcting ∆F508-CFTR folding defects and enhancing its functionality, leading to marked improvements in respiratory function among CF patients (Keating et al., 2018). However, despite the success of Trikafta, there are persistent challenges. ΔF508-CFTR remains ubiquitinated even in the presence of Trikafta, and prolonged administration of IVA destabilizes ΔF508-CFTR on the cell surface (Cholon et al., 2014; Veit et al., 2014). Furthermore, the maturation achieved with Trikafta results in a considerably shorter half-life of ΔF508-CFTR compared to the wild-type CFTR (Capurro et al., 2021; Taniguchi et al., 2022). In a clinical investigation, it was observed that Trikafta treatment led to a notable reduction in systemic pro-inflammatory cytokines and a restoration of circulating immune cell composition. However, despite a 12-month monitoring period, these enhancements did not reach the levels observed in individuals categorized as healthy controls (Sheikh et al., 2023). Hence, there exists potential for further enhancement in Trikafta’s efficacy, which may yield improved clinical therapeutic outcomes.

In this study, we conducted a ligand-based *in silico* screening of CFTR modulators utilizing machine learning techniques based on publicly available CFTR modulator data. Through this method, we identified a novel CFTR corrector termed FR3. Our research demonstrates that FR3 exhibits the ability to stabilize the ∆F508-NBD1 and enhance the cell surface expression of ∆F508-CFTR in cell culture models. Notably, FR3 appears to operate through a distinct mechanism from the currently approved CFTR modulator. Combining FR3 with TEZ/ELX/IVA showed a synergistic effect, enhancing TEZ/ELX/IVA efficacy. Additionally, our findings indicate that FR3 functions as a CFTR stabilizer, effectively preventing the degradation of mature ∆F508-CFTR even in the presence of TEZ/ELX/IVA. Furthermore, FR3 exhibited promising results by improving the PM expression of ∆Y490-ABCB1 and rare CFTR mutants when used in conjunction with TEZ/ELX/IVA. These observations may highlight the potential of FR3 as a lead compound for addressing diseases stemming from misfolded ABC transporters.

## 2 Materials and methods

### 2.1 *In silico* screening

#### 2.1.1 Training set

Active molecules (there are 605) come from PubChem assay 743267 (https://pubchem.ncbi.nlm.nih.gov/bioassay/743267). Known CF drugs (Lumacaftor (LUM), Ivacaftor (IVA), Tezacaftor (TEZ), Elexacaftor (ELX)), plus all known correctors (C1 to C18, plus C4172), potentiators (P1 to P10) and blockers (B1 to B8) from the CF foundation compound program were also added as actives to this training set. Inactive molecules (there are 342,350) come from PubChem assay 720511 (https://pubchem.ncbi.nlm.nih.gov/bioassay/720511). This is the primary screen, while we took active molecules from a confirmatory screen, as a precautionary measure. All molecules were standardized using Francis Atkinson’s standardizer (https://github.com/flatkinson/standardiser). Duplicate molecules and molecules failing standardization were excluded from the training set.

#### 2.1.2 Machine-learning models

Classification model 1: all molecules were encoded using the “signature molecular descriptor” (Faulon et al., 2003) with height two bonds. We used two bonds because the performance of the model in terms of early recovery was a little bit better than using one bond. This is a counted, unfolded fingerprint. On this dataset, it takes into account 40,063 features. To accelerate modeling and screening, the total number of molecules was capped to 65,000 (about 100 inactives per active molecule), using all actives but only a random partition from the inactive molecules. As machine-learning method, we used a prototype software developed in the lab using Kernel Density Estimate to rank-order molecules and at the same time obtain an applicability domain for the model (Berenger and Yamanishi, 2020). The biweight kernel was used. Median kernel bandwidth over 11 modeling experiments: 0.69. AUC in a 10 folds cross validation experiment: 0.81. Platt scaling (Platt, 1996) parameters: A = −359.43 B = 4.29.Those parameters allow to transform raw predicted scores into binding probabilities.

A second model was trained, so that predictions do not rely on a single molecular encoding and machine-learning combination. Classification model 2: a long ECFP6 fingerprint was used (recommended by (OBoyle and Sayle, 2016)). It is a folded, uncounted fingerprint with 16384 bits. Liblinear’s L2-regularized logistic regression was used (Fan et al., 2008), with class probabilities. Five models were trained on balanced random bootstraps extracted from the training set. Final predictions are the average of those five models. Optimization parameter C was left to its default value (1.0) because it was found that on this dataset, bagging is more reliable than trying to optimize C. During tests, our bag of five models showed a test set AUC of 0.83.

#### 2.1.3 Production virtual screen

We screened the 4,326,442 purchasable compounds from Kishida chemicals (http://www.kishida.co.jp/). All compounds were standardized, then encoded using the molecular encoding required by each model. Final selection of compounds: only the top-scoring 500 compounds found by averaging the predictions from both models were kept. Those compounds were filtered so that a given Bemis-Murcko scaffold (Bemis and Murcko, 1996) is seen only once (diversity selection). This reduced the selection to 61 compounds. The selection was further shrunk to the top 20 scoring compounds. Those compounds were annotated by finding the nearest active molecule from the training set. Selected compounds found similar to an active molecule from the training set were removed. The 20 molecules left had a predicted binding probability p in [0.35:0.91] according to model 1 and *p* ≥ 0.88 according to model 2.

### 2.2 Reagents and chemicals

The following chemicals were used: DMSO (Sigma-Aldrich, St Louis, MO, Cat# D2650), MG-132 (Cayman Chemical, Ann Arbor, MI, Cat# 10012628), Lumacaftor (Cayman Chemical, Cat# 22196), Tezacaftor (Selleck Chemicals, Houston, TX, Cat# S7059), Elexacaftor (Selleck Chemicals, Cat# S8851), Ivacaftor (Chemscene LLC, Monmouth Junction, NJ, Cat# CS-0497), cycloheximide (CHX, FUJIFILM Wako Pure Chemical Corporation, Osaka, Japan, Cat# 3720991), doxycycline (dox, FU-JIFILM Wako Pure Chemical Corporation, Cat# 049-31121), glycerol (FU-JIFILM Wako Pure Chemical Corporation, Cat# 075-00616), cyclosporin A (CLP-A, FU-JIFILM Wako Pure Chemical Corporation, Cat# 031-24931), E-4031 (Selleck Chemicals, Cat# S6627). Hit compounds from the *in silico* screening and the FR3 analog compounds are listed in Supplementary Table S1.

### 2.3 Cell lines and cell culture

Parental CFBE41o- (CFBE), CFBE tet-on cells stably expressing ∆F508-CFTR-HRP, ∆F508-R1S-CFTR-HRP, ∆F508-R1070W-CFTR-HRP, ∆F508-CFTR-3HA, HBH-∆F508-CFTR-3HA, HBH-∆F508-R1S-CFTR-3HA, HBH-∆F508-R1070W-CFTR-3HA or ∆F508-CFTR-3HA and YFP-F46L/H148Q/I152L were cultured as previously (Rabeh et al., 2012; Okiyoneda et al., 2013; Okiyoneda et al., 2018). CFBE tet-on cells stably expressing inducible ΔF508-CFTR-3HA-NLuc were established by lentivirus transduction as previously (Taniguchi et al., 2022) and were cultured in minimal essential medium (MEM, FUJIFILM Wako Pure Chemical Corporation) supplemented with 10% FBS and 3 μg/mL puromycin. The CFTR expressions in CFBE cells were induced by treating them with 1 μg/mL dox for 4 days. BEAS-2B cells stably expressing CFTR variants-HiBiT, ∆Y490-ABCB1-HiBiT, or G601S-hERG-HiBiT were established by lentivirus transduction as previously (Taniguchi et al., 2022) and were cultured in Dulbecco’s Modified Eagle Medium (DMEM) (FUJIFILM Wako Pure Chemical Corporation) supplemented with 10% FBS and 10 μg/mL blasticidin S. The HiBiT tag was introduced to the extracellular region of CFTR (4^th^ extracellular loop), ABCB1 (1^st^ extracellular loop) (Kamada et al., 2023) or hERG (1^st^ extracellular loop). The hERG-HiBiT was produced by replacing the extracellular HA tag (Apaja et al., 2013) with the HiBiT tag by PCR-based mutagenesis.

### 2.4 Measurement of PM expression of CFTR, ABCB1, and hERG

Cell-surface expression of CFTR-HRP in CFBE cells on 96 well plates was measured after the addition of the HRP substrate (SuperSignal West Pico PLUS, ThermoFisher) as previously (Okiyoneda et al., 2018; Taniguchi et al., 2022). Cell-surface expression of CFTR-HiBiT, ABCB1-HiBiT, and hERG-HiBiT in BEAS-2B cells on 96 well plates was measured using the Nano Glo HiBiT Extracellular system (Promega, Madison, WI) as previously (Taniguchi et al., 2022). For low-temperature rescues, CFBE and BEAS-2B cells were incubated at 26°C and 30°C, respectively, for 48 h followed by 1-h incubation at 37°C to induce unfolding. In BEAS-2B cells, 26°C treatment was found to be cytotoxic; therefore, 30°C treatment was performed instead. For the TEZ/ELX/IVA rescue, cells were incubated with 3 μM TEZ, 1 μM ELX, and 1 μM IVA at 37°C for 48 h. BEAS-2B-HiBiT cells were treated with 2 mM sodium butyrate (NaB) for 48 h before analysis. The luminescent signal was measured using the Luminoskan and Varioskan Flash microplate reader (ThermoFisher Scientific, Waltham, MA).

### 2.5 Western blotting

Cells were solubilized in a RIPA buffer supplemented with 1 mM PMSF, 5 μg/mL leupeptin, and 5 μg/mL pepstatin A. Equal amounts of proteins in cell lysate were analyzed by a Western blot using anti-HA (16B12, BioLegend, San Diego, CA, Cat# 901515), anti-HiBiT (Promega, Cat# N7200) antibodies or HRP-Neutravidin (HRP-NA, ThermoFisher, Cat #A2664) as previously (Okiyoneda et al., 2010; Okiyoneda et al., 2018). Western blots were visualized using a SuperSignal West Pico PLUS Chemiluminescent Substrate (ThermoFisher) or ImmunoStar Zeta (FUJIFILM Wako Pure Chemical Corporation, Osaka, Japan) and analyzed by FUSION Chemiluminescence Imaging System (Vilber Bio Imaging, France). The staining of Ponceau S (Sigma-Aldrich, St Louis, MO) was used as a loading control.

### 2.6 Halide-sensitive YFP quenching assay

The ΔF508-CFTR function assay by halide-sensitive YFP fluorescence quenching was performed as described previously (Okiyoneda et al., 2018). The PM expression of ΔF508-CFTR in CFBE-tet-on-∆F508-CFTR-3HA/YFP-H148Q/I152L/F46L cells were induced by treatment of 1 μM ELX or TEZ/ELX/IVA (3 μM TEZ, 1 μM ELX, 1 μM IVA) for 48 h at 37°C. Fluorescence was measured using a VICTOR Nivo multimode microplate reader (PerkinElmer, Waltham, MA) with a dual syringe pump (excitation/emission 500/535 nm). The CFTR was activated by cAMP cocktails (20 µM forskolin (FUJIFILM Wako Pure Chemical Corporation), 0.5 mM 3-isobutyl-1-methyl-xanthine (IBMX, FUJIFILM Wako Pure Chemical Corporation), 0.5 mM 8-(4-chlorophenylthio)-adenosine-3′, 5′-cyclic monophosphate (CPT-cAMP, Santa Cruz Biotechnology, Santa Cruz, CA), and 0.1 mM genistein (FUJIFILM Wako Pure Chemical Corporation) for 57 s before the rapid addition of PBS-iodide. The fluorescence was recorded continuously (200 ms per point) for 3 s (baseline) and for 32 s after the rapid addition of 100 µL PBS-iodide, in which NaCl was replaced with NaI. Quenching rates were calculated by fitting the YFP fluorescence decay with a one-phase exponential decay function using GraphPad Prism 8 (GraphPad Software).

### 2.7 Quantitative real-time PCR

The mRNA isolation and real-time quantitative PCR were performed as described previously (Hinata et al., 2023). The following primers were used; CFTR FW primer 5′- AGT​GGA​GGA​AAG​CCT​TTG​GAG​T -3′, CFTR RV primer 5′-ACA​GAT​CTG​AGC​CCA​ACC​TCA-3′, GAPDH FW primer 5′- CAT​GAG​AAG​TAT​GAC​AAC​AGC​CT-3′, GAPDH RV primer 5′- AGT​CCT​TCC​ACG​ATA​CCA​AAG​T-3’.

### 2.8 CFTR-NLuc degradation assay

The CFTR-NLuc degradation assay was performed as previously (Taniguchi et al., 2022). CFBE tet-on ΔF508-CFTR-3HA-NLuc cells were seeded onto 96 well plates and treated with 1 μg/mL dox for 4 days. PM expression of ΔF508-CFTR was induced by treatment of TEZ/ELX/IVA (3 μM TEZ, 1 μM ELX, 1 μM IVA) for 48 h at 37°C. Cells were loaded with Nano-Glo^®^ Endurazine (Promega) in CO_2_ independent medium (ThermoFisher) for 3 h. When measuring the degradation of mature ΔF508 CFTR, 100 μg/mL Cycloheximide (CHX) was treated to minimize the immature ΔF508-CFTR during Nano-Glo Endurazine substrate loading. After 3 h of loading, the CFTR-NLuc luminescence was recorded continuously (10 min per point) in the presence of CHX at 37°C using a Luminoskan microplate reader (ThermoFisher). The degradation rate and half-life of ΔF508-CFTR-NLuc were calculated by fitting with a one-phase exponential decay function using GraphPad Prism 8 (GraphPad Software).

### 2.9 DSF thermal shift assay

The melting temperature (T_m_) of recombinant ∆F508-NBD1 isolated from *E. Coli* was measured as previously (Okiyoneda et al., 2013). Differential Scanning Fluorimetry (DSF) scans of 4.95 µM His_6_-sumo-ΔF508-1S NBD1 in HEPES buffer (150 mM NaCl, 5 mM MgCl_2_, and 100 mM HEPES, pH 7.5) were performed using a LightCycler^®^ 480 System II (Roche, Switzerland) qPCR instrument in the presence of 5X SYPRO Orange (ThermoFisher Scientific, Waltham, MA). The LightCycler^®^ 480 Software (Roche) was used to calculate the first derivate of the resulting melting curve, with the steepest point of the slope being the T_m_.

### 2.10 Short-circuit current measurement

The ΔF508-CFTR function assay by short-circuit current measurement was performed as described previously (Hinata et al., 2023). CFBE tet-on ΔF508-CFTR-3HA cells were plated on fibronectin-coated, 12 mm Snapwell filters (Corning, Corning, NY, United States) at a density of 1 × 10^5^ cells/cm^2^ and treated with 1 μg/mL dox for 4 days. PM expression of ΔF508-CFTR was induced by treatment of TEZ/ELX/IVA (3 μM TEZ, 1 μM ELX, 1 μM IVA) for 48 h at 37°C. The polarized epithelia (≥5 days after confluence) were mounted in Ussing chambers (U-2500, Warner Instruments, Holliston, MA, United States), bathed in Krebs-bicarbonate Ringer, and continuously bubbled with 95% O_2_ and 5% CO_2_. To impose a Cl^−^ gradient, Cl^−^ was replaced by gluconate in the apical compartment. To functionally isolate the apical membranes, the basolateral PM was permeabilized with 100 µM amphotericin B (FUJIFILM Wako Chemicals, Japan), and the epithelial sodium channel was inhibited with 100 μM amiloride (TCI, Japan). CFTR activity was stimulated by apical forskolin (10 μM, FUJIFILM Wako Chemicals), followed by the addition of CFTR inhibitor 172 (Inh172, 20 μM, Santa Cruz Biotechnology, Dallas, TX, USA) to determine CFTR-specific apical Cl^−^ current. Measurements were performed at 37°C and recorded using a current-clamp amplifier (CEZ-9100, Nihon Kohden, Japan) and PowerLab 2/26 system (ADInstruments, New Zealand).

### 2.11 Statistical analysis

For quantification, data from at least 3 independent experiments were used where the data are expressed as means ± SE. Statistical significance was assessed by either a two-tailed unpaired Student’s t-test, a one-way analysis of variance (ANOVA) with Dunnett’s multiple comparison test, or a two-way ANOVA with Holm-Sidak multiple comparison tests was performed using GraphPad Prism 8 (GraphPad Software Inc., San Diego, CA). A *p*-value <0.05 was defined as statistically significant.

## 3 Results

### 3.1 *In silico* screening identified FR3 as an ΔF508-CFTR ligand

**FIGURE 1:**
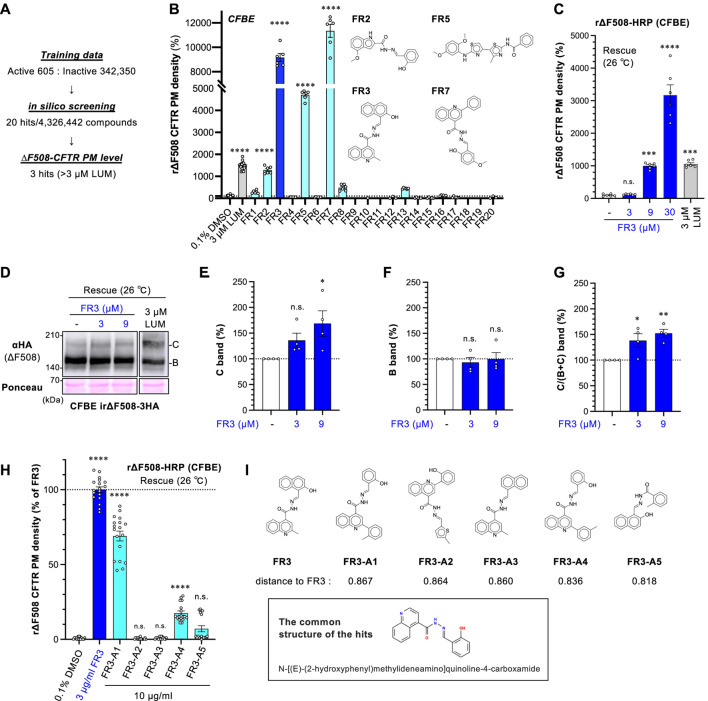
FR3 increases the PM level of ∆F508-CFTR. **(A)** Overview of the *in silico* virtual screening. **(B)** The secondary screening of 20 hit compounds (10 μg/mL). The PM level of rΔF508-CFTR-HRP in CFBE Tet-on cells was measured. Cells were treated with compounds for 48 h at 26°C, followed by a 1-h incubation at 37°C (*n* = 6). The structure of hit compounds was shown. **(C)** Dose-dependent effect of FR3 on rΔF508-CFTR-HRP PM level in CFBE Tet-on cells (*n* = 6). Compounds were treated as **(B) (D–G)** Western blotting measured the expression of rΔF508-CFTR-3HA in CFBE Tet-on cells. The maturation of ∆F508-CFTR was induced by 26°C incubation for 48 h, followed by a 1-h incubation at 37°C. Cells were treated with FR3 for the last 24 h or 3 µM LUM for 48 h before cell lysis. Mature (band C, E) and immature (band B, F) forms of rΔF508-CFTR-3HA were quantified by densitometry (*n* = 4). The C/(B + **(C)** band ratio was also quantified (G, *n* = 4). **(H)** The effects of FR3 analog on the PM level of rΔF508-CFTR-HRP in CFBE Tet-on cells (*n* = 18). Cells were treated as **(D) (I)** The structure of FR3 analog compounds and the common structure of the active compounds. Distance to FR3 was calculated using an unfolded-counted molecular fingerprint. Statistical significance was assessed by one-way ANOVA with Dunnett’s multiple comparison tests. Data represent mean ± SE. *p < 0.05, **p < 0.01, ****p < 0.0001, n.s., not significant.

To discover new CFTR correctors, we performed an *in silico* screening of 4,326,442 compounds available from Kishida chemicals using a ligand-based approach based on the chemical structures of existing CFTR-improving drugs. For this screen, we selected 605 active molecules and 342,350 inactives from Pubchem as training data to construct a machine-learning model. As a result of the *in silico* screening, we pinpointed 20 compounds with a high probability of binding to CFTR (Figure 1A). In a subsequent screening phase, these 20 compounds were tested on human CF bronchial epithelial cell lines (CFBE cells) that stably expressed ∆F508-CFTR-HRP. We were simultaneously conducting the low-temperature incubation (26°C rescue) and FR3 treatment, aiming for an add-on effect of simultaneous treatment and a marked improvement in the PM expression. Using the HRP assay, only three compounds (FR3, FR5, and FR7) demonstrated stronger enhancement of the cell surface expression of r∆F508-CFTR-HRP compared to the well-known CFTR corrector LUM (Figure 1B). Further investigations were directed towards FR3, given that FR5 was reported as a CFTR corrector (WO 2009051909, (Ye et al., 2010)), and FR7 was potentially associated with CFTR according to patent information (WO 2008/121877). The identification of two CFTR-related compounds, FR5 and FR7, through our approach, serves as a robust validation of the effectiveness of our methodology in discovering novel CFTR modulators.

We confirmed that FR3 increased the cell surface expression of r∆F508-CFTR-HRP in a dose-dependent manner (Figure 1C). The effect of FR3 at higher concentrations, such as 9 and 30 µM for 48 h treatment, resulted in greater improvement of the PM level compared to LUM (Figure 1C). However, it's important to note that these FR3 effects may be overestimated due to the observed cytotoxicity associated with 9 μM and 30 µM FR3 treatment for 48 h at 26°C (Supplementary Figure S1A). Therefore, Western blot analysis was conducted with 24-h treatment at 26°C, which showed minimal cytotoxicity (Supplementary Figure S1A). Western blot analysis revealed that treatment with FR3 for 24 h specifically augmented the mature form of rΔF508-CFTR in a concentration-dependent manner, leaving the immature form unaffected (Figures 1D–F). Furthermore, the ratio of the mature form to the total CFTR level increased following FR3 treatment (Figure 1G). These results suggest that FR3 may function as a CFTR ligand, enhancing the maturation and/or stability of the mature form of ΔF508-CFTR.

We assessed several commercially available FR3 analogs to identify potential compounds with enhanced efficacy. Among the tested analogs, only FR3-A1 and FR3-A4 exhibited significant effects on the cell surface level of r∆F508-CFTR-HRP in CFBE cells, albeit to a lesser extent than FR3 itself (Figures 1H,I). Notably, the shared structural motif among these active compounds was identified as N-[(E)-(2-hydroxyphenyl) methylideneamino] quinoline-4-carboxamide (Figure 1I). This common structure suggests a potential pharmacophore for targeting and modifying the activity of these compounds.

### 3.2 FR3 enhances functional PM expression of ΔF508-CFTR by clinically utilized CFTR modulators

**FIGURE 2:**
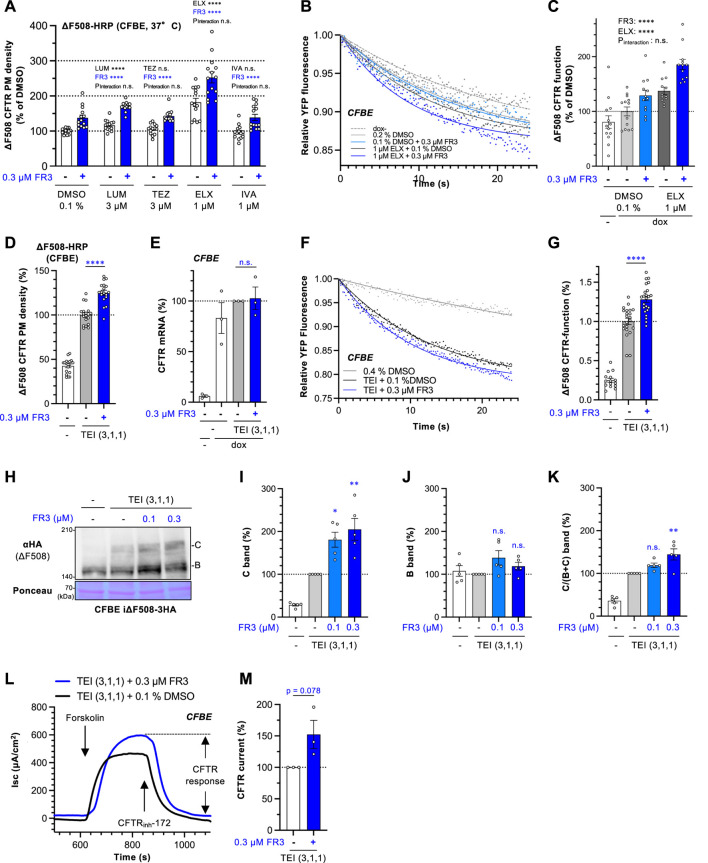
FR3 enhances the ∆F508-CFTR rescue effect of TEZ/ELX/IVA. **(A)** The impact of the combined application of FR3 alongside a CFTR modulator was measured by the ΔF508-CFTR-HRP assay in CFBE Tet-on cells treated with compounds indicated at 37°C for 48 h (*n* = 14). **(B, C)** Representative traces **(B)** of the YFP fluorescence and quantification of the initial YFP quenching rate **(C)** as ΔF508-CFTR function in CFBE cells treated with 0.3 μM FR3 with or without 1 μM ELX at 37°C for 48 h (*n* = 12–13). **(D)** The effect of the combination of FR3 and TEZ/ELX/IVA (TEI) on the PM level of ΔF508-CFTR-HRP in CFBE Tet-on cells. Cells were treated with 0.3 μM FR3 and TEI (3 µM TEZ, 1 µM ELX, 1 µM IVA) at 37°C for 48 h (*n* = 18). **(E)** The mRNA level of exogenous ΔF508-CFTR-3HA in CFBE Tet-on cells treated as D (*n* = 3). **(F, G)** The effect of the combination of FR3 and TEI on the ΔF508-CFTR function. Representative traces **(F)** of the YFP fluorescence and quantification of the initial YFP quenching rate **(G)** as ΔF508-CFTR function in CFBE cells treated with 0.3 μM FR3 with or without TEI (3 µM TEZ, 1 µM ELX, 1 µM IVA) at 37°C for 48 h (*n* = 12–13). **(H–K)** The effect of the combination of FR3 and TEI on the ΔF508-CFTR level in CFBE Tet-on cells was measured by Western blotting. Compounds were treated at 37°C for 48 h. Mature (band C, I) and immature (band B, J) forms of ΔF508-CFTR-3HA were quantified by densitometry (n = 5). The C/(B + **(C)** ratio was also quantified **(K)**, *n* = 5). **(L, M)** Apical ΔF508-CFTR-3HA current (Isc) in CFBE Tet-on cells was measured after sequential addition of 10 μM forskolin and 100 μM genistein, followed by CFTR inhibition with 20 μM CFTRinh-172. FR3 (0.3 µM) and TEI (3 µM TEZ, 1 µM ELX, 1 µM IVA) were treated at 37°C for 48 h before Isc measurement. The effect of FR3 on the ΔF508-CFTR Isc was calculated and expressed as % of the Isc in FR3-untreated cell. **(M)**, *n* = 3) Statistical significance was assessed by two-way ANOVA with Holm-Sidak multiple comparison tests **(A, C)**, a two-tailed unpaired Student’s t-test **(D, E, G, M)**, or one-way ANOVA with Dunnett’s multiple comparison tests **(I–K)**. Data represent mean ± S.E. *p < 0.05, **p < 0.01, ***p < 0.001, ****p < 0.0001, n.s., not significant.

To understand how FR3 operates, we investigated whether it could enhance the effects of clinically available CFTR modulators. The HRP assay revealed that CFTR correctors LUM and TEZ, as well as CFTR potentiator IVA, had minimal impact on the cell surface levels of ∆F508-CFTR-HRP in CFBE cells. In contrast, the second-generation CFTR corrector ELX showed a modest increase in CFTR levels at the PM (Figure 2A). In contrast to the treatment at 26°C, FR3 treatment at higher concentrations, such as 3 μM, was more cytotoxic at 37°C (Supplementary Figure S1B). Thus, we opted for a lower concentration (0.3 µM) of FR3 for combined treatment with CFTR modulators. Notably, the HRP assay demonstrated that FR3’s effect was independent of CFTR modulators and resulted in an additional increase in the PM levels of ∆F508-CFTR-HRP alongside CFTR modulators. In particular, FR3 and ELX additively increased the PM level, resulting in the highest improvement among the conditions we tested (Figure 2A). This suggests a distinct mechanism of action compared to conventional CF modulators (Figure 2A).

To ascertain whether FR3 enhanced the functional channel of ∆F508-CFTR, we conducted the halide-sensitive YFP quenching assay (Okiyoneda et al., 2018). While FR3 or ELX alone marginally improved the channel function, their combination showed an additive increase in the functional ∆F508-CFTR at the PM (Figures 2B,C). Considering FR3’s unique actions from existing CFTR modulators, we anticipated potential synergy with TEZ/ELX/IVA. Indeed, the HRP assay demonstrated that 0.3 µM FR3 significantly elevated the PM levels of ∆F508-CFTR when administered in conjunction with TEZ/ELX/IVA in CFBE cells (Figure 2D). This increased CFTR level did not result from an increase in mRNA levels (Figure 2E). Furthermore, the YFP quenching assay indicated that FR3 significantly augmented the functional ∆F508-CFTR channel at the PM upon TEZ/ELX/IVA treatment (Figures 2F,G). Moreover, the Western blot analysis corroborates these observations, indicating that in the presence of TEZ/ELX/IVA, FR3 specifically augmented the mature form of ∆F508-CFTR while showing a marginal impact on the immature form (Figures 2H–K). We also examined the impact of FR3 on CFTR function in polarized CFBE cells expressing ∆F508-CFTR using short-circuit current measurement (Isc). CFTR function was quantified from the CFTR inh-172 sensitive current. As expected, FR3 treatment tended to increase the ∆F508-CFTR current following treatment with TEZ/ELX/IVA (Figures 2L, M).

### 3.3 FR3 stabilizes ΔF508-NBD1 and the mature form of ΔF508-CFTR

**FIGURE 3:**
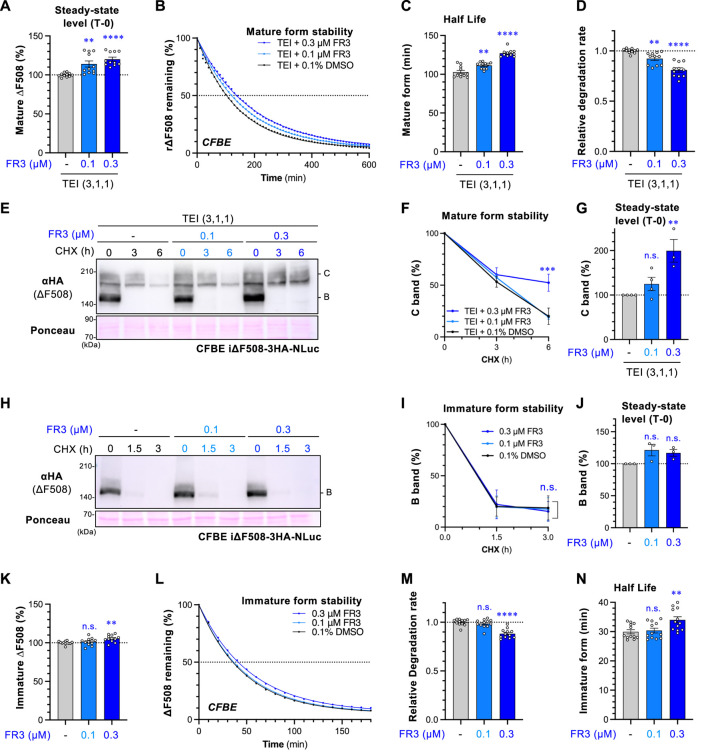
FR3 attenuates the degradation of ∆F508-CFTR. **(A)** The steady-state level of mature ∆F508-CFTR-NLuc in CFBE cells was assessed following treatment with TEI (3 µM TEZ, 1 µM ELX, 1 µM IVA) for 48 h, with FR3 added for the last 24 h at 37°C. **(B–D)** The effect of FR3 on the degradation of mature ∆F508-CFTR-NLuc in CFBE cells treated as **(A)**. Representative traces of mature ∆F508-CFTR-NLuc elimination were shown **(B)**. The half-life **(C)** and degradation rate **(D)** of mature ΔF508-CFTR-NLuc were calculated by fitting the degradation curve (*n* = 11). **(E–J)** Western blotting with CHX chase measured the stability of mature ΔF508-CFTR-3HA-NLuc **(E–G)** and the immature form **(H–J)** in CFBE Teton cells treated as Fig.3A. Cells were treated with TEI to analyze the mature form. The stability and steady-state levels of both the mature (band C, F, G) and immature (band B, I, J) ΔF508-CFTR were quantified by densitometry (*n* = 3). **(K)** Steady-state level of immature ∆F508-CFTR-NLuc in CFBE cells treated with FR3 for 24 h at 37°C. **(L–N)** The effect of FR3 on the degradation of immature ∆F508-CFTR-NLuc in CFBE cells treated as **(K)**. Representative traces of immature ∆F508-CFTR-NLuc elimination were shown **(L)**. The degradation rate **(M**) and half-life **(N)** of immature ΔF508-CFTR-NLuc were calculated by fitting the degradation curve (*n* = 12). Statistical significance was assessed by either one-way ANOVA with Dunnett’s multiple comparison tests **(A, C, D, G, J, K, M, N)** or two-way ANOVA with Holm-Sidak multiple comparison tests **(F, I)**. Data represent mean ± S.E. **p < 0.01, ***p < 0.001, ****p < 0.0001, n.s., not significant.

To investigate if FR3 enhances the stability of mature ΔF508-CFTR, we utilized a ΔF508-CFTR-NLuc assay in CFBE cells (Taniguchi et al., 2022). NLuc was fused with the C-terminus of CFTR in the cytoplasmic region, and its luminescence signal reflects the CFTR-NLuc amount in cells. Treatment with FR3 increased the steady-state level of ∆F508-CFTR-NLuc upon TEZ/ELX/IVA treatment (Figure 3A). The CHX chase revealed a continuous reduction in the NLuc signal, with a half-life of approximately 100 min, consistent with the reported half-life of mature ∆F508-CFTR (Taniguchi et al., 2022; Kamada et al., 2023) (Figures 3B,C). Interestingly, FR3 treatment, in a concentration-dependent manner, weakly but significantly reduced the degradation of mature ∆F508-CFTR induced by TEZ/ELX/IVA treatment, thereby extending its half-life (Figures 3B–D). These findings were supported by Western blot analysis in CHX chase experiments, where 0.3 µM FR3 prevented the degradation of mature ΔF508-CFTR after TEZ/ELX/IVA treatment (Figures 3E,F). Consequently, FR3 increased the steady-state level of mature ΔF508-CFTR (Figure 3G), consistent with the enhanced expression at the PM (Figure 2D) and improved function (Figure 2G). However, FR3 had no discernible impact on the steady-state level and degradation of immature ΔF508-CFTR in the absence of TEZ/ELX/IVA (Figures 3H–J). Similarly, the ∆F508-CFTR-NLuc assay indicated that 0.3 µM FR3 minimally increased the steady-state level (Figure 3K) and half-life of immature ∆F508-CFTR by reducing ERAD (Figures 3L–N).

**FIGURE 4:**
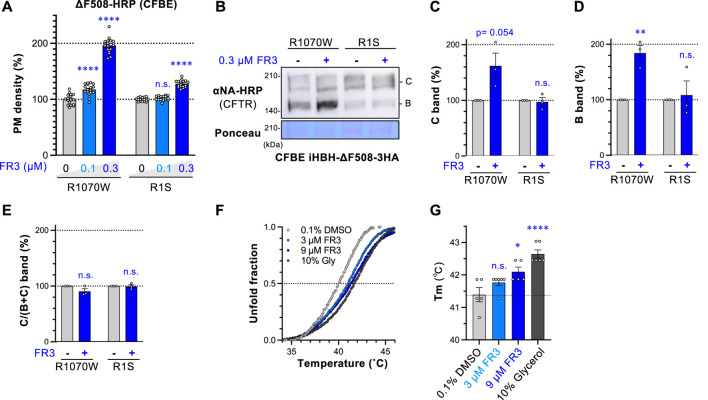
FR3 stabilizes ∆F508-NBD1 and corrects the defective expression of ∆F508-R1070W-CFTR. **(A)** The PM levels of ΔF508-R1070W-CFTR-HRP and ΔF508-R1S-CFTR-HRP in CFBE Teton cells treated with FR3 at 37°C for 48 h (*n* = 15). **(B–E)** Western blotting measured the expression of HBH-ΔF508-R1070W-CFTR-3HA and HBH-ΔF508-R1S-CFTR-3HA in CFBE Teton cells treated with FR3 as **(A)**. The mature (band C, C) and immature (band B, D) forms of the CFTR variants were quantified by densitometry (*n* = 3). The C/(B + **(C)** ratio was also calculated **(E)**, *n* = 3. **(F, G)** The melting curve **(F)** and temperature (T_m_, **(G)** of purified His_6_-sumo-ΔF508-NBD1 in the presence of compounds were measured *in vitro* (*n* = 5–6). Statistical significance was assessed by either one-way ANOVA with Dunnett’s multiple comparison tests **(A, G)** or a two-tailed unpaired Student’s t-test **(C–E)**. Data represent mean ± S.E. *p < 0.05, **p < 0.01, ****p < 0.0001, n.s., not significant.

FR3 potentiates the effect of LUM or TEZ (Figure 2A) and might therefore stabilize ∆F508-NBD1. This is because stabilizing NBD1 enhances the effects of LUM or TEZ (Okiyoneda et al., 2013; Veit et al., 2020). To test this hypothesis, we assessed FR3’s effect on ∆F508-CFTR with R1070W or R1S suppressor mutations. R1070W and R1S mutations are known to correct ∆F508-CFTR conformational defects in the NBD1-MSD2 interface and NBD1, respectively (Rabeh et al., 2012; Okiyoneda et al., 2013). The PM density measurement revealed that FR3 significantly increased the PM level of ∆F508-CFTR-R1070W-HRP while showing a modest effect on ∆F508-CFTR-R1S-HRP (Figure 4A). In line with this, Western blotting showed that FR3 increased both immature and mature forms of ∆F508-CFTR-R1070W, but not ∆F508-CFTR-R1S (Figures 4B–E). These results suggest that FR3 could primarily correct the instability of ∆F508-NBD1. To confirm this hypothesis, we measured FR3’s impact on the thermal stability of recombinant ∆F508-NBD1 using the differential scanning fluorimetry (DSF) thermal shift assay (Rabeh et al., 2012). The melting temperature (T_m_) of ∆F508-NBD1-1S was approximately 41.5°C, higher than previously reported (Rabeh et al., 2012), likely due to the presence of the His_6_-sumo tag (Figures 3T–U). As anticipated, FR3 at a higher concentration (9 µM) significantly increased the T_m_, although its effect was weaker than that of the chemical chaperone glycerol (Okiyoneda et al., 2013) (Figures 4F,G). These findings strongly suggest that FR3 stabilizes ΔF508-NBD1 through a mechanism distinct from clinically used CFTR modulators, thus promoting the maturation and stabilization of ΔF508-CFTR in conjunction with TEZ/ELX/IVA.

### 3.4 FR3 rescues the PM expression of ΔY490-ABCB1 and CFTR rare mutants

**FIGURE 5:**
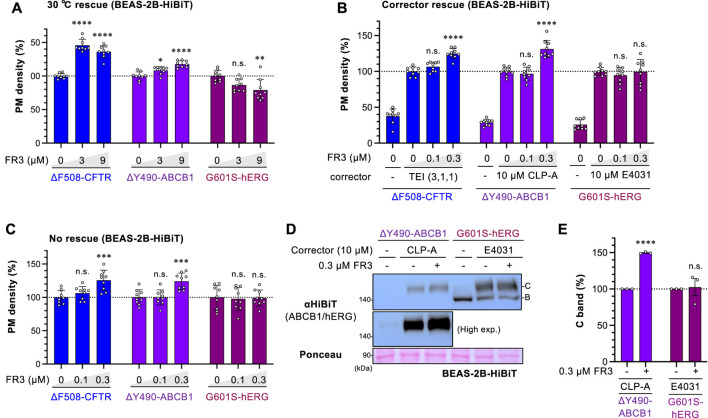
Effect of FR3 on ΔY490-ABCB1. **(A–C)** The PM levels of ΔF508-CFTR-HiBiT, ΔY490-ABCB1-HiBiT, and G601S-hERG-HiBiT in BEAS-2B cells (*n* = 9). **(A)** Cells were incubated at 30°C for 48 h and treated with FR3 for the last 24 h at 30°C, followed by a 1-h incubation at 37°C before analysis. **(B, C)** Cells were treated with lower concentrations of FR3 for 48 h in the presence **(B)** or absence of a respective pharmacological chaperone (corrector) at 37°C **(C)**. **(D, E)** Western blotting showing the expression of ΔY490-ABCB1-HiBiT and G601S-hERG-HiBiT in BEAS-2B cells treated with FR3 as 5B. The mature (band C) forms of ΔY490-ABCB1-HiBiT and G601S-hERG-HiBiT were quantified by densitometry **(E)**, *n* = 3. Statistical significance was assessed by one-way ANOVA with Dunnett’s multiple comparison tests **(A–C)** or a two-tailed unpaired Student’s t-test **(E)**. Data represent mean ± S.E. *p < 0.05, **p < 0.01, ***p < 0.001, ****p < 0.0001, n.s., not significant.

To explore whether FR3 non-selectively corrects the PM expression of abnormal membrane proteins, we examined its impact on ΔY490-ABCB1 and G601S-hERG in BEAS-2B human bronchial epithelial cells. ΔY490-ABCB1, akin to ∆F508-CFTR, bears a mutation in the cytoplasmic NBD1 of ABCB1, leading to misfolding and retention in the ER (Hoof et al., 1994; Gnann et al., 2004; Nakatsukasa and Brodsky, 2008). On the other hand, G601S-hERG is a mutation in the hERG potassium channel associated with inherited long QT syndrome (Furutani et al., 1999; Delisle et al., 2003), predominantly retained in the ER due to misfolding (Smith et al., 2011). We introduced the HiBiT tag to the extracellular region of ABCB1 or the hERG mutant to measure the cell surface level. To accurately evaluate FR3’s effect, we measured the PM level of ΔY490-ABCB1-HiBiT and G601S-hERG-HiBiT under three different conditions. Upon low-temperature rescue, which facilitates the PM expression of thermally unstable proteins like ∆F508-CFTR (Denning et al., 1992), FR3 slightly increased the PM level of ΔY490-ABCB1-HiBiT, albeit to a lesser extent than observed with ∆F508-CFTR (Figure 5A). Additionally, FR3 elevated the PM level of ΔY490-ABCB1-HiBiT after cyclosporin-A (CLP-A) treatment, a pharmacological chaperone for ABCB1 (Loo and Clarke, 1997) (Figure 5B), and without any rescue treatment (Figure 5C). However, FR3 did not enhance the PM level of G601S-hERG-HiBiT, irrespective of the rescue treatment (Figures 5A–C). E4031, an hERG inhibitor, acts as a pharmacological chaperone aiding the PM arrival of the misfolded hERG channel (Zhou et al., 1999). Western blotting aligned with our findings, demonstrating that while FR3 increased the mature ΔY490-ABCB1, it did not affect G601S-hERG under their respective pharmacological rescue conditions (Figures 5D,E). These outcomes suggest that FR3 selectively facilitates the PM arrival of specific types of misfolded membrane proteins. Given that both ∆F508-CFTR and ΔY490-ABCB1 exhibit equivalent NBD1 instabilities, FR3 likely stabilizes their NBD1, thereby enhancing PM expression.

**FIGURE 6:**
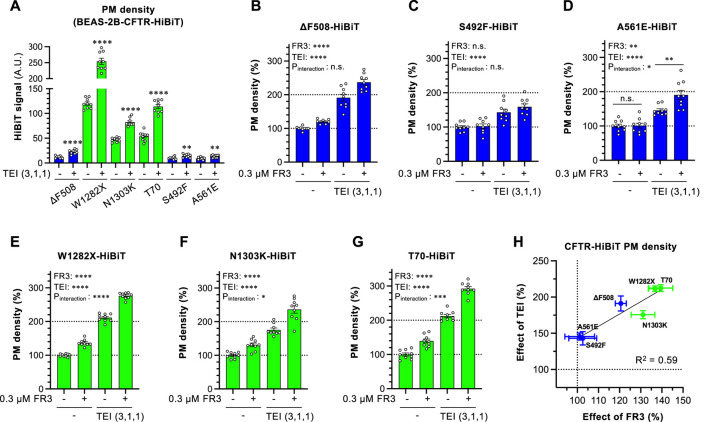
Effect of FR3 on CF-associated CFTR mutants. **(A)** Effect of TEZ/ELX/IVA (TEI) on the PM level of CF-associated CFTR mutants harboring the extracellular HiBiT tag in BEAS-2B cells. Cells were treated with or without TEI (3 µM TEZ, 1 µM ELX, 1 µM IVA) at 37°C for 48 h (*n* = 7–9). **(B–G)** PM levels of ΔF508 **(B)**, S492F **(C)**, A561E **(D)**, W1282X **(E)**, N1303K **(F)**, T70 **(G)** CFTR-HiBiT in BEAS-2B cells treated with FR3 and TEI for 48 h at 37°C (*n* = 7–9). **(H)** Correlation between the effects of FR3 and TEI on the PM levels of CFTR mutants evaluated in G-L. The correlation coefficient is shown as R-square. Statistical significance was assessed by a two-tailed unpaired Student’s t-test **(A)** or two-way ANOVA with Holm-Sidak multiple comparison tests **(B–G)**. Data represent mean ± S.E. *p < 0.05, **p < 0.01, ***p < 0.001, ****p < 0.0001, n.s., not significant.

Extending our analysis beyond ∆F508-CFTR, we assessed FR3’s effect on other disease-causing CFTR mutants in BEAS-2B cells. Among the CFTR mutants studied, W1218X-, N1303K-, and T70-CFTR were expressed at the PM without TEZ/ELX/IVA, while S492F- and A561E-CFTR showed marginal PM levels similar to ∆F508-CFTR (Figure 6A). As reported previously (Han et al., 2018; Phuan et al., 2019; McKee et al., 2023), TEZ/ELX/IVA increased PM levels in all CFTR mutants analyzed (Figure 6A). FR3 alone did not enhance the PM expression of ∆F508-, S492F-, or A561E-CFTR. However, in the presence of TEZ/ELX/IVA, the membrane expression of A561E-CFTR improved, similar to ∆F508-CFTR (Figures 6B–D). Notably, FR3 did not increase the PM level of S492F-CFTR, regardless of TEZ/ELX/IVA treatment (Figure 4H). Conversely, FR3 alone significantly elevated the PM level of W1282X-, N1303K-, or T70-CFTR, and in synergy with TEZ/ELX/IVA, enhanced PM levels (Figures 6E–G). The analysis examining the correlation between the effects of FR3 and TEZ/ELX/IVA demonstrated a strong relationship, particularly highlighting FR3’s heightened effectiveness against CFTR mutants that are readily responsive to TEZ/ELX/IVA (Figure 6H). Particularly noteworthy is FR3’s high efficacy against CFTR mutants (e.g., N1303K, W1282X, T70), sharing characteristics of class 6 mutants exhibiting PM instability (Veit et al., 2016).

## 4 Discussion

In this study, we successfully identified a novel CFTR modulator, FR3, through a ligand-based *in silico* screening. To date, prior research has primarily focused on structure-based *in silico* screening techniques utilizing existing CFTR modulator binding sites or targeting abnormal folding sites (Kalid et al., 2010; Odolczyk et al., 2013; Orro et al., 2021; Fossa et al., 2022). However, the utilization of ligand-based *in silico* screening incorporating CFTR modulator information has been relatively infrequent. Our rediscovery of two potential CFTR modulators, FR5 (WO 2009051909) and FR7 (an analog listed in WO 2008/121877) through our approach validates the efficacy of our methodology in identifying CFTR modulators. This successful identification underscores the credibility and potential of our strategy in uncovering compounds capable of modulating CFTR function.

FR3 appears to function as a CFTR corrector, demonstrated by its ability to elevate the PM levels of ∆F508-CFTR at 37°C. Notably, FR3 exhibited an additive effect in increasing the PM levels of r∆F508-CFTR when used in combination with LUM, TEZ, or ELX. This suggests that the mode of action of FR3 might not overlap with these CFTR correctors. While the precise binding sites of FR3 remain unclear, our *in vitro* assays indicated that FR3 directly interacts with ∆F508-NBD1, enhancing its thermal stability. Although a previous study showed ELX binding to and stabilizing NBD1 (Veit et al., 2020), recent cryo-electron microscopy structural analysis revealed ELX, along with TEZ and IVA, binding to MSDs (Fiedorczuk and Chen, 2022). Therefore, FR3 might possess distinct binding sites, such as NBD1, differing from clinically used CFTR modulators. This distinct binding capacity may contribute to its additive improvement in both the PM levels and function of ∆F508-CFTR in combination with other modulators. FR3 corrected the PM expression of ∆Y490-ABCB1, which shares equivalent NBD1 conformational defects (Hoof et al., 1994) but did not impact G601S-hERG, lacking NBD. This observation supports the notion that FR3 specifically binds and stabilizes the NBD. Although further investigations are warranted, FR3’s efficacy might extend to other misfolded ABC transporter mutants exhibiting NBD abnormalities (Loo et al., 2005; Rudashevskaya et al., 2014; Hegde et al., 2017).

Remarkably, FR3 demonstrated robust stabilization of the mature form of ∆F508-CFTR in the presence of TEZ/ELX/IVA in our cell culture model. Previous studies have indicated that despite TEZ/ELX/IVA treatment, ΔF508-CFTR remains susceptible to ubiquitination and degradation, resulting in comparatively lower stability at the PM when compared to the wild-type CFTR (Capurro et al., 2021; Taniguchi et al., 2022). Notably, prior research revealed that glycerol, as a chemical chaperone increasing the thermal stability of NBD1, augmented the PM stability of ∆F508-CFTR (Okiyoneda et al., 2013). Thus, the NBD1 stabilization prompted by FR3 might contribute to stabilizing the mature form of ∆F508-CFTR. Interestingly, the combination of FR3 with TEZ/ELX/IVA did not notably affect the immature form of ∆F508-CFTR. This finding suggests the intriguing possibility that TEZ/ELX/IVA correction of CFTR’s conformational defects might be adequate to evade the ERQC checkpoint. However, it appears that despite TEZ/ELX/IVA treatment, the peripheral QC system might still recognize residual CFTR conformational defects. In this context, FR3 seems to function as a CFTR stabilizer, counteracting peripheral degradation mediated by ubiquitin ligases (Okiyoneda et al., 2018; Taniguchi et al., 2022).

Unexpectedly, FR3 exhibited favorable effects on W1282X-, N1303K-, and T70-CFTR mutants, despite the absence of mutations in the NBD1. Nevertheless, previous studies employing limited proteolysis analysis indicated the potential for alterations in the NBD1 conformation within N1303K-CFTR and T70-CFTR (Benharouga et al., 2001; Du and Lukacs, 2009). This suggests the presence of structural abnormalities in the NBD1 regions of these CFTR mutants, which might render them more responsive to the effects of FR3. These CFTR mutants share characteristics categorized as class 6 mutants (Veit et al., 2016) and exhibit some degree of PM expression even without TEZ/ELX/IVA. Earlier studies have suggested that CFTR mutants demonstrating moderate PM expression and residual function tend to exhibit heightened responsiveness to CFTR modulators (Han et al., 2018; McKee et al., 2023). Corresponding with these findings, the impact of TEZ/ELX/IVA or FR3 on the PM level was notably more pronounced in these mutants. Significantly, FR3 independently elevated the PM levels of W1282X-, N1303K-, and T70-CFTR, likely by inhibiting peripheral degradation. Hence, FR3 may hold promise as a seed compound for the development of CFTR stabilizers aimed at treating CF patients carrying class 6 CFTR mutations, although further investigation using primary cell culture models is necessary.

## Data Availability

The datasets presented in this study can be found in online repositories. The names of the repository/repositories and accession number(s) can be found in the article/Supplementary Material.
