# Supplementary material for: Ligand-based virtual-screening identified a novel CFTR ligand which improves the defective cell surface expression of misfolded ABC transporters
Source: Front Pharmacol. 2024 Apr 11;15:1370676. doi: 10.3389/fphar.2024.1370676 (PMC11043560; doi:10.3389/fphar.2024.1370676)
Supplement: Supplementary file 1 [file DataSheet1.PDF]

Supplemental Information

Ligand-based virtual-screening identified a novel CFTR ligand which improves the defective cell surface expression of misfolded ABC transporters

Shogo Taniguchi[1], Francois Berenger[2], Yukako Doi[1], Ayana Mimura[1], Yoshihiro Yamanishi[3], Tsukasa Okiyoneda[1*]

[1]Department of Biomedical Sciences, School of Biological and Environmental Sciences, Kwansei Gakuin University, Hyogo 669-1337, Japan.
[2]Graduate School of Frontier Sciences, The University of Tokyo, 5-1-5 Kashiwa-no-ha, Kashiwa, Chiba 277-8561, Japan
[3]Department of Complex Systems Science, Graduate School of Informatics, Nagoya University, Graduate School of Informatics, Nagoya University, Chikusa, Nagoya 464-8601, Japan

* Correspondence: Tsukasa Okiyoneda, t-okiyoneda@kwansei.ac.jp

Figure S1. The cytotoxic effect of FR3 at 26°C or 37°C incubation in CFBE cells
(A) CFBE Tet-on cells stably expressing ΔF508-CFTR-HRP were treated with FR3 at the indicated concentrations for the last 24 or 48 hours at 26 °C, followed by a 1-hour incubation at 37 °C. For comparison, DMSO or LUM was treated for 48 hours at 26 °C instead of FR3.
(B) CFBE Tet-on cells stably expressing ΔF508-CFTR-HRP were treated with FR3 at the indicated concentrations for the last 24 or 48 hours at 37 °C. For comparison, DMSO or LUM was treated for 48 hours at 37 °C instead of FR3.
The cytotoxic effects were evaluated using the alamarBlue assay according to the manufacturer's instructions (ThermoFisher Scientific). Data represent mean ± S.E (n = 6-12).

Figure S1

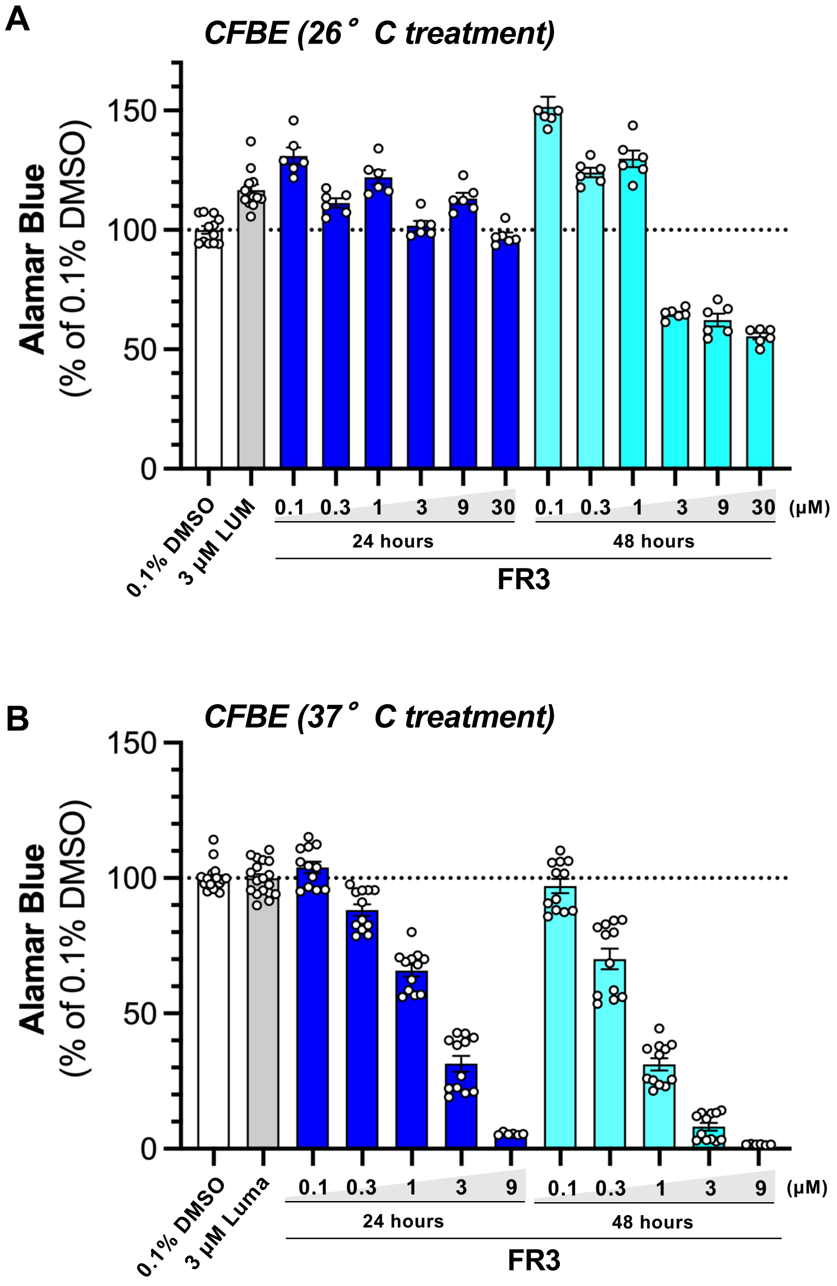
A
CFBE (26° C treatment)
Alamar Blue
(% of 0.1% DMSO)
150
100
50
0
0.1% DMSO
3 µM LUM
0.1 0.3 1 3 9 30
24 hours
0.1 0.3 1 3 9 30
48 hours
(µM)
FR3
B
CFBE (37° C treatment)
Alamar Blue
(% of 0.1% DMSO)
150
100
50
0
0.1% DMSO
3 µM Luma
0.1 0.3 1 3 9
24 hours
0.1 0.3 1 3 9
48 hours
(µM)
FR3
